# Thromboelastometry analysis of thrombocytopenic dengue patients: a cross-sectional study

**DOI:** 10.1186/s12879-017-2204-4

**Published:** 2017-01-19

**Authors:** Felipe Maia de Toledo Piza, Thiago Domingos Corrêa, Alexandre Rodrigues Marra, João Carlos Campos Guerra, Roseny dos Reis Rodrigues, Andrea Aparecida Rocco Villarinho, Valdir Fernandes de Aranda, Sandra Christina Pereira Lima Shiramizo, Maria Roza de Jesus de Lima, Esper Georges Kallas, Alexandre Biasi Cavalcanti

**Affiliations:** 1 Department of Intensive Care Medicine, Hospital Israelita Albert Einstein, Av. Albert Einstein, 627/701, 5° andar, São Paulo, CEP: 05651-901 Brazil; 2 Hospital das Clinicas da Faculdade de Medicina da Universidade de São Paulo, São Paulo, Brazil

## Abstract

**Background:**

Dengue virus infection (DVI) is a prevalent and potentially fatal viral disease associated with coagulopathy. So far, the coagulation profile of DVI patients with thrombocytopenia has not been assessed through a viscoelastic test such as rotational thromboelastometry. We aimed to describe the prevalence and characteristics of coagulation abnormalities in dengue fever outpatients with thrombocytopenia, addressed by both rotational thromboelastometry and conventional coagulation tests.

**Methods:**

This was a cross-sectional study conducted between April 6^th^ and May 5^th^ 2015 in São Paulo, Brazil during a dengue outbreak. Thromboelastometry (ROTEM®) and the conventional coagulation tests prothrombin time (PT), international normalized ratio (INR), activated partial thromboplastin time (aPTT), thrombin time (TT), platelet count and fibrinogen levels were performed in 53 patients with DVI and thrombocytopenia.

**Results:**

Despite a median interquartile range (IQR) platelet count of 77 (63–88) x 10^9^/L in DVI patients, conventional coagulation tests and plasma fibrinogen levels were within the normal range. Subjects demonstrated hypocoagulability in 71.7% (38/53) in INTEM and 54.7% (29/53) in EXTEM DVI patients. FIBTEM analyses detected only 5.7% (3/53) with hypocoagulability among this population. The median (IQR) clotting time (CT), clot formation time (CFT) and maximum clot firmness (MCF) on INTEM were, respectively, 177 (160–207) sec, 144 (108–178) sec and 48 (42–52) mm. On EXTEM, median (IQR) CT, CFT and MCF were, respectively, 69 (65–78) sec, 148 (126–198) sec and 49 (44–55) mm. Median (IQR) MCF on FIBTEM was 15 (13–18) mm.

**Conclusion:**

Thromboelastometry impairment is highly prevalent in DVI patients with thrombocytopenia, particularly in INTEM and EXTEM analyses, while standard coagulation tests are normal in this setting. Clinical implications remain to be established.

## Background

Dengue is by far the most incident human arbovirus disease [1], with over 2.5 billion people living in high-risk transmission areas [2]. The World Health Organization (WHO) estimates 50–100 million of dengue virus infections (DVI) per year, resulting in 500,000 hospitalizations and 20,000 deaths worldwide [1, 2].

Dengue hemorrhagic fever (DHF) represents a severe clinical presentation of DVI and is characterized by the presence of varying degrees of hemostatic disorders [3, 4]. Intense and amplified cytokine release, along with the complement activation, result in endothelial dysfunction, platelet destruction and consumption of coagulation factors, which may lead to a life threatening disseminate intravascular coagulation (DIC) [5, 6]. Indeed, blood coagulation disorders are commonly observed in patients with DHF and dengue shock syndrome [6, 7].

Many studies have assessed the coagulation system in DVI through conventional coagulation tests such as the prothrombin time (PT), international normalized ratio (INR), thrombin time (TT), and activated partial thromboplastin time (aPTT) [3, 6, 8, 9]. Nevertheless, conventional coagulation tests were validated to monitor vitamin K antagonists and heparin therapy [10, 11]. Although conventional coagulation tests have not been validated to predict and/or to guide therapy in acute (acquired) hemorrhage, they have been widely used for this purpose [10]. Conventional coagulation tests results may take a few hours to be completed and reported, they track the complexity of hemostatic impairment poorly, and most frequently, they reflect late coagulopathy disorders [10–13].

Rotational thromboelastometry (ROTEM®) is a point of care test that promptly provides (5–30 min) information about the dynamics of clot formation, stabilization and dissolution, reflecting the in vivo hemostasis at the bedside [12]. ROTEM provides more clinically useful and reliable information than the conventional coagulation tests in critically ill patients [13, 14], yielding a graphical presentation of fibrin polymerization process, involving fibrinogen and platelet function, and fibrinolysis [13].

To our knowledge, no study has evaluated the coagulation profile of patients with DVI with rotational thromboelastometry. Therefore, we aimed at describing the prevalence of coagulation abnormalities addressed by both thromboelastometry and conventional coagulation tests in cases of dengue fever outpatients with thrombocytopenia. Additionally, we evaluated the correlation between conventional coagulation tests and thromboelastometry in this population of patients.

## Methods

### Study design and setting

This was a cross-sectional study conducted during a DVI outbreak in São Paulo, Brazil between April 6^th^ and May 5^th^ 2015. This study was approved by the University of São Paulo Institutional Review Board [*Comissão de Ética para Análise de Projetos e Pesquisas* (CAPPesq), approval number: 0652/09]. All patients provided informed consent prior inclusion in this study.

### Participants

**Fig. 1:**
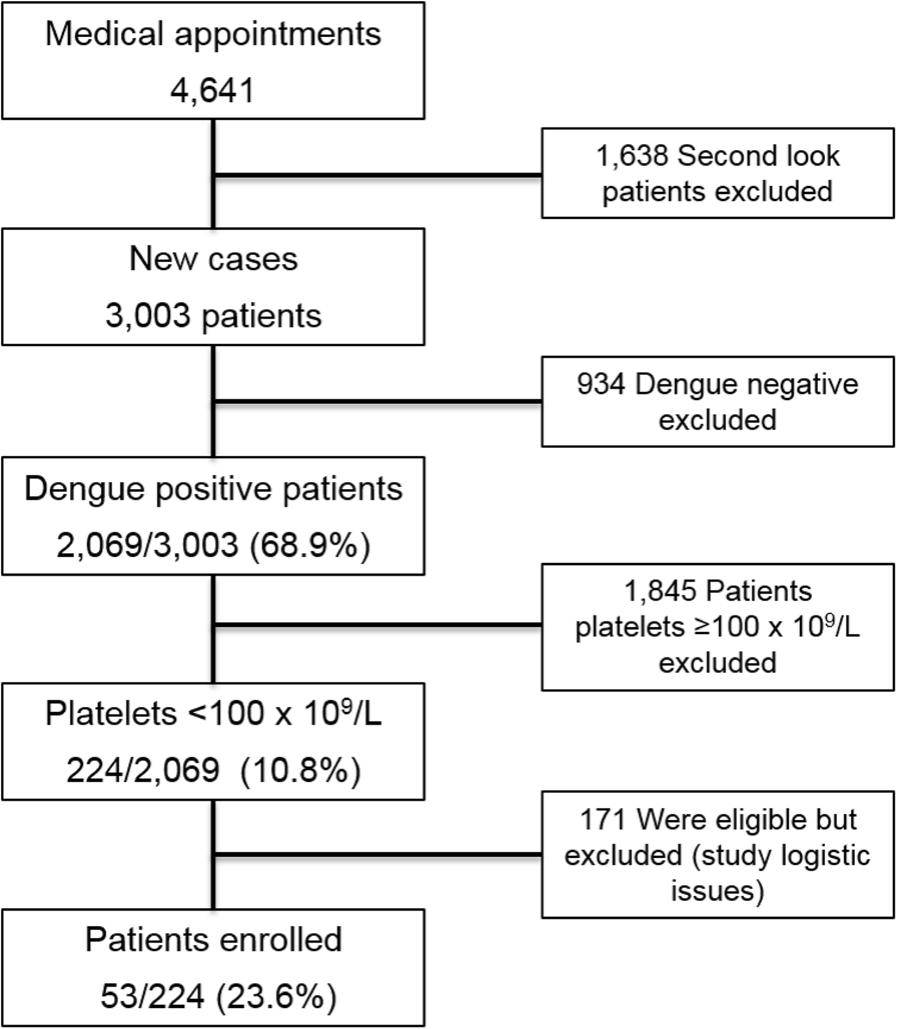
Patients’ flow diagram

Patients were screened at an outpatient clinic established in a neighborhood in the city of São Paulo with high incidence of DVI. Patients with at least 24 h of fever (axillary temperature >37.8 °C), in addition to a positive dengue virus (DV) specific immunoglobulin IgM/IgG or non-structural protein-1 (NS1) antigen rapid test (DENGUE DUO Bioeasy®, Kyonggi-Do, South Korea) and platelet count <100 x 10^9^/L were consecutively included in this study (Fig. 1).

Exclusion criteria included pregnancy, presence of oncologic or hematologic disorders, solid or bone marrow transplantation, secondary thrombocytopenia, previous known coagulopathy, chronic hepatitis B or C, chronic renal failure and use of anti-platelet therapy or vitamin K antagonists.

### Dengue virus infections triage, support and blood sampling

**Fig. 2:**
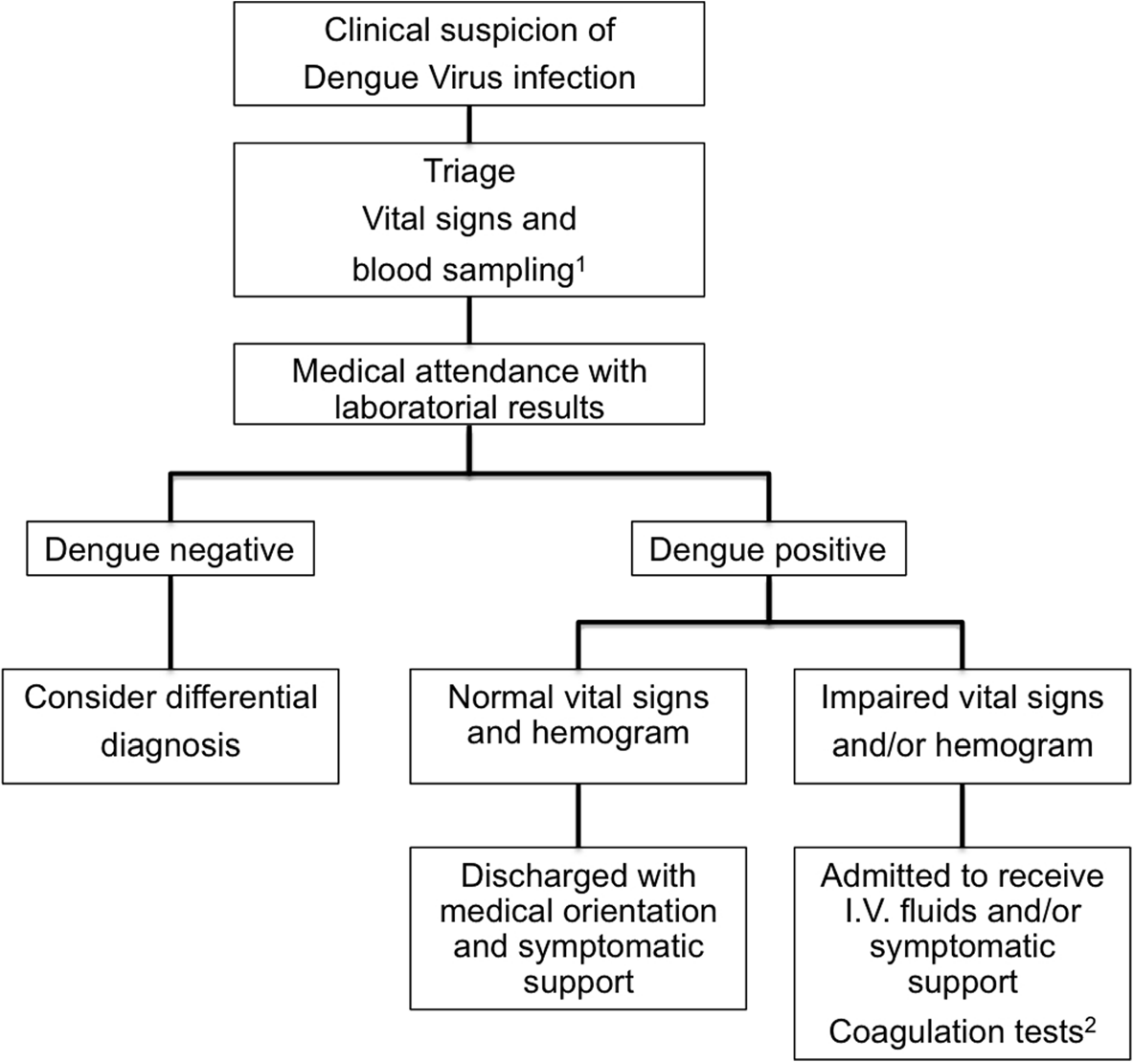
Patient’s flow chart attendance during dengue virus infection outbreak. Legend: ^1^: blood samples for detection of dengue virus specific IgM/IgG and NS1 antigen, hemoglobin, hematocrit, white blood cell and platelet count. ^2^: in the 53 patients with confirmed dengue virus infection and thrombocytopenia (platelet count <100 x 10^9^/L), conventional coagulation tests (PT, TT, INR and aPTT), serum fibrinogen level, plasma d-dimer levels and rotational thromboelastometry (ROTEM®) were performed

All patients who visited the outpatient clinic had their clinical history taken to obtain information on length of time, type and severity of symptoms and vital signs analysis (arterial blood pressure, axillary temperature and heart rate) (Fig. 2). Demographic data, comorbidities, clinical presentation, vital signs and the need for hospital admission were recorded.

Once DVI was suspected, blood samples were collected and tested for detection of DV-specific IgM/IgG and NS1 antigen (Fig. 2). Additionally, hemoglobin, hematocrit, white blood cell and platelets count were performed (XS-1000i™; Sysmex Roche®, Kobe, Japan).

Patients with confirmed DVI and arterial hypotension [systolic blood pressure (SBP) <90 mmHg], fever (temperature >37.8 °C), tachycardia [heart rate (HR) >100 bpm) and/or hemoconcentration (hematocrit >50% for men or >48% for women) were admitted for intravenous (I.V.) fluid administration and/or symptomatic support (Fig. 2). In 53 patients with confirmed DVI and thrombocytopenia (platelet count <100 x 10^9^/L), conventional coagulation tests (PT, TT, INR and aPTT) (STA Compact; Stago®, Asnières-sur-Seine, France), serum fibrinogen level (Clauss method) (STA Compact; Stago®, Asnières-sur-Seine, France), plasma d-dimer level (STA Compact; Stago®, Asnières-sur-Seine, France) and rotational thromboelastometry (ROTEM®; Pentapharm Co., Munich, Germany) were performed.

### Thromboelastometry

Thromboelastometry (ROTEM) assesses the viscoelastic properties of the clot and provides information on the speed of coagulation initiation, kinetics of clot formation, clot strength and breakdown [14, 15]. Blood samples of 3 ml were collected in citrate tubes (3.2%; Sarsted®, Wedel, Germany) and processed within a period of three hours for thromboelastometry analysis. The analyses were performed by pipetting 300 μl of citrated whole blood and 20 μl of 0.2 M calcium chloride with specific activators into a plastic cup [16]. Measurement of coagulation in ROTEM was performed after the vertical immersion of a plastic pin into the blood sample. The pin rotates slowly backwards and forwards through an angle of 4.75°. By generation of the first fibrin filaments between the pin and the wall of the test cup, the rotational range of the pin is reduced [13]. The increased restriction of the pin’s movement is transferred to a graphical display, a plot that shows changes in the viscoelastic properties of the clot over time [15].

The following parameters were recorded during ROTEM analysis: clotting time [CT; seconds (sec)], which represents the beginning of the test until a clot firmness of 2 mm is detected, clot formation time (CFT; sec), until a clot firmness of 20 mm has been reached, alpha angle (degrees) represents the slope (tangent) between a CT of 2 mm and CFT of 20 mm, amplitude 10 (A10; mm), which represents the clot amplitude 10 min after the beginning of clotting and maximum clot firmness (MCF; mm), which represents the greatest amplitude of the thromboelastometric trace and reflects the “strength” of the clot [13–16]. We performed an extrinsically activated thromboelastometric test (EXTEM), a test that uses rabbit brain thromboplastin as an activator; an intrinsically activated thromboelastometric test (INTEM), a test that uses ellagic acid as an activator and a fibrin-based thromboelastometric test (FIBTEM), a test that assesses the fibrin-based clot using both extrinsic activation and addition of cytochalasin D to inhibit platelets’ contribution to clot formation [13, 15].

Impaired ROTEM (hypocoagulability) was defined as a clot formation speed above the upper limit of the reference interval (CT INTEM >246 s or CT EXTEM >74 s) and/or (CFT INTEM >100 s or CFT EXTEM >148 s) and/or a MCF below the lower limit of the reference interval (INTEM MCF <52 mm or EXTEM MCF <49 mm) and/or alpha angle below the lower limit of the reference interval (INTEM alpha angle <70° or EXTEM alpha angle <63°) [13, 15]. Finally, impaired ROTEM on FIBTEM was defined as a MCF <9 mm [16]. Normal ROTEM was considered if CT, CFT, MCF and alpha angle were within the reference ranges (INTEM CT: 137–246 s, CFT: 40–100 s, alpha angle: 70–83° and MCF: 52–72 mm; EXTEM CT: 42–74 s, CFT: 46–148 s, alpha angle: 63–83° and MCF: 49–71 mm; FIBTEM MCF: 9–25 mm) [15, 16].

### Statistical analysis

Categorical variables were presented as absolute and relative frequencies. Continuous variables were presented as mean values and standard deviation (SD) or median values with interquartile ranges (IQR) in case of non-normal distribution (tested by the Kolmogorov-Smirnov test). Continuous variables were compared using independent *t* test or Mann–Whitney *U* test in case of non-normal distribution.

Two-tailed tests were used and when *p* < 0.05, the test was considered statistically significant. The SPSS™ (IBM SPSS Statistics for Windows, Version 21.0. Armonk, NY: IBM Corp) and GraphPad Prism version 6.0 (GraphPad Software, La Jolla, California, USA) were used for statistical analyses.

## Results

From April 6^th^ to May 5^th^ 2015, 4641 medical appointments were scheduled at the outpatient clinic. From those, 3003 were first consultations (Fig. 1). Dengue virus infections were confirmed in 68.9% (2069/3003) of patients while 10.8% (224/2069) of patients had DVI and thrombocytopenia. Out of those, fifty-three patients [53/224 (23.6%)] were consecutively enrolled in this study (Fig. 1).

The main characteristics of DVI patients included in this study are shown in Table 1. The median age of DVI patients was 32 (IQR, 21–43) years and approximately 60% were male. The majority of DVI patients [31/42 (73.8%)] had no significant previous medical history. Most DVI patients presented with fever and headache and were hemodynamically stable (Table 1). Onset of symptoms was acute (median, 4 days; IQR, 2–5), which is consistent with predominance of NS1 positive results [51/53 (96.2%)]. Bleeding manifestations such as epistaxis, gingivorrhagia, hematemesis, hematuria and melena were reported in only 14.3% (7/49) of patients (Table 1). A total of 5.8% (3/52) of patients required hospitalization.

**Table 1:** Main characteristics of participating patients. Values represent % (n/total) or median (interquartile range [IQR])

Characteristics	Values^a^
Age, years	32 (21–43)
Gender, male	60.4 (32/53)
Comorbidities	
None	73.8 (31/42)
Hypertension	16.7 (7/42)
HIV infection	2.4 (1/42)
Prostatic hyperplasia	4.8 (2/42)
Dyspepsia	2.4 (1/42)
Clinical presentation	
Fever	100.0 (53/53)
Headache	83.0 (44/53)
Rash	36.8 (14/38)
Vomiting	25.5 (13/51)
Bleeding manifestations^b^	14.3 (7/49)
Dehydration^c^	11.3 (6/53)
Axillary temperature	37.0 (36.1-37.7)
Systolic blood pressure	127 (115–133)
Diastolic blood pressure	80 (73–86)
Heart rate	93 (82–102)
Serologic diagnosis	
NS1	96.2 (51/53)
IgM	20.8 (11/53)
IgG	3.8 (2/53)
Prior DV infection^d^	9.8 (5/51)
Yellow Fever vaccination^e^	18.9 (7/37)
Days after onset of symptoms	4 (2–5)
Need of hospitalization^f^	5.8 (3/52)

*HIV* Human Immunodeficiency Virus, *NS1* non-structural protein-1, *IgM* Immunoglobulin M, *IgG* Immunoglobulin G; ^a^ Totals may not sum to 53, owing to missing values; ^b^ epistaxis, gingivorrhagia, hematemesis, hematuria and melena; ^c^ clinical signs in mucosa and skin assessed by medical attendant; ^d^ considered if patient acknowledge a prior diagnosis stated by medical service. ^e^: considered if patient acknowledge as a positive previous vaccination status, ^f^: Unknown in one patient (lost to follow-up)

### Laboratorial characteristics

The main laboratorial characteristics of DVI patients are shown in Table 2. All DVI patients with thrombocytopenia [median (IQR) platelets count: 76 (62–88) x10^9^/L] had normal coagulation tests such as PT, TT, INR and aPTT (Table 2).

**Table 2:** Laboratory and conventional coagulation tests result in dengue virus infection patients. Values are presented as median and interquartile range

Characteristics	
Hemoglobin (g/dL)	15.1(14.2-16.1)
Hematocrit (%)	42.9 (40.6-45.6)
White Blood Cells (x10^3^/uL)	3.1 (2.7-4.5)
Neutrophil (%)	42.3 (31.0-48.0)
Neutrophil (x10^3^/uL)	1.4 (1.0-1.8)
Platelets (x 10^9^/L)	76 (62–88)
Prothrombin time (sec)	100 (90–100)
INR	1.0 (1.0-1.1)
Thrombin Time (sec)	18.2 (17.0-19.5)
aPTT (sec)	28.9 (26.0-32.5)
Fibrinogen (g/dl)	290 (267–323)
D-dimer (ng/ml)	1330 (800–1840)

*INR* international normalized ratio, *aPTT* activated partial thromboplastin time

### Thromboelastometry

The INTEM and EXTEM analysis were abnormal in, respectively, 71.7% (38/53) and 54.7% (29/53) of DVI patients while FIBTEM was normal in 94.3% (50/53) of DVI patients (Table 3). DVI patients with impaired (hypocoagulability) ROTEM in INTEM and EXTEM assays exhibited higher CFT, lower MCF and alpha angle than DVI patients with a normal ROTEM (Table **3**).

**Table 3:** Rotational thromboelastometry (ROTEM**®**) parameters in dengue virus infection patients. Values represent median (IQR)

Characteristics	All	Normal	Impaired	*P* value*
INTEM, % (n/total)	100.0 (53/53)	28.3 (15/53)	71.7 (38/53)	
Clotting time (sec)	177 (160–207)	166 (158–179)	180 (161–214)	0.114^b^
Clot formation time (sec)	144 (108–178)	98 (90–106)	166 (131–220)	<0.001^b^
Maximum clot firmness (mm)	48 (42–52)	53 (52–55)	45 (41–49)	<0.001^a^
Alpha angle (degrees)	69 (63–73)	74 (72–74)	66 (62–69)	<0.001^a^
Amplitude 10 (mm)	41 (37–45)	48 (47–50)	37 (33–42)	<0.001^a^
EXTEM, % (n/total)	100.0 (53/53)	45.3 (24/53)	54.7 (29/53)	
Clotting time (sec)	69 (65–78)	68 (61–74)	74 (66–84)	0.044^a^
Clot formation time (sec)	148 (126–198)	126 (114–140)	197 (163–269)	<0.001^b^
Maximum clot firmness (mm)	49 (44–55)	54 (52–56)	44 (40–48)	<0.001^a^
Alpha angle (degrees)	68 (63–72)	72 (68–74)	65 (56–69)	<0.001^b^
Amplitude 10 (mm)	41 (35–46)	46 (44–48)	36 (31–39)	<0.001^a^
FIBTEM, % (n/total)	100.0 (53/53)	94.3 (50/53)	5.7 (3/53)	
Maximum clot firmness (mm)	15 (13–18)	16 (14–18)	7 (7–8)	0.004^b^

*: Comparisons between normal and impaired groups. p values with (^a^) independent *t* test and (^b^) Mann–Whitney *U* test

Hypocoagulability was found in 28.6% (2/7) of DVI patients with minor bleeding manifestations in EXTEM and in 57.1% (4/7) patients in INTEM. Out of three patients who required hospitalization, two (66.6%) exhibited hypocoagulability in INTEM and one (33.3%) on EXTEM.

### Conventional coagulation tests versus thromboelastometry

Comparisons between thromboelastometry and conventional coagulation tests are shown in Table 4. Compared to DVI patients with normal INTEM, DVI patients presenting with impaired INTEM exhibited lower platelet count, INR and plasma fibrinogen levels (Table 4). Dengue virus infection patients presenting with impaired EXTEM exhibited lower plasma fibrinogen levels and higher d-dimer, while platelets count, INR and aPTT did not differ between the groups (Table 4). Finally, conventional coagulation tests did not differ between DVI patients with normal or abnormal FIBTEM (Table 4).

**Table 4:** Rotational thromboelastometry (ROTEM**®**) analysis and conventional coagulation tests. Values represent median (IQR)

Characteristics	Normal	Impaired	*P* value
INTEM, % (n/total)	28.3 (15/53)	71.7 (38/53)	
Platelets (x 10^9^/L)	90 (77–94)	70 (57–84)	0.005^a^
INR	1.1 (1.0-1.3)	1.0 (1.0-1.1)	0.034^b^
aPTT (sec)	28.2 (26.0-31.8)	29.0 (26.0-33.3)	0.867^b^
Fibrinogen (g/dl)	321 (290–355)	278 (267–311)	0.021^b^
D-dimer (ng/ml)	950 (320–1510)	1410 (950–1900)	0.076^b^
EXTEM, % (n/total)	45.3 (24/53)	54.7 (29/53)	
Platelets (x 10^9^/L)	82 (68–91)	69 (57–83)	0.052^a^
INR	1.0 (1.0-1.1)	1.0 (1.0-1.1)	0.390^b^
aPTT (sec)	30.3 (25.0-32.2)	28.1 (26.6-32.8)	0.816^b^
Fibrinogen (g/dl)	308 (278–354)	278 (264–300)	0.006^a^
D-dimer (ng/ml)	950 (705–1500)	1600 (1220–1990)	0.008^b^
FIBTEM, % (n/total)	94.3 (50/53)	5.7 (3/53)	
Platelets (x 10^9^/L)	77 (62–88)	71 (64–95)	0.758^b^
INR	1.0 (1.0-1.1)	1.1 (1.0-1.1)	0.427^b^
aPTT (sec)	28.5 (26.0-31.8)	33.3 (26.7-33.4)	0.254^b^
Fibrinogen (g/dl)	291 (267–323)	278 (214–315)	0.520^b^
D-dimer (ng/ml)	1295 (790–1820)	1840 (1500–1980)	0.223^b^

*INR* international normalized ratio and *aPTT* activated partial thromboplastin time. *p* values with (^a^) independent *t* test and (^b^) Mann–Whitney *U* test

## Discussion

This study demonstrated that DVI patients with thrombocytopenia frequently exhibited hypocoagulability assessed by thromboelastometry while conventional coagulation tests (PT, TT, INR and aPTT) and plasma fibrinogen levels remained within reference range.

Our findings contrasted previous retrospective studies, which demonstrated that prolonged coagulation times are frequent and strongly associated with bleeding manifestations in DVI thrombocytopenic patients [8, 9, 17, 18]. For instance, Wills and colleagues demonstrated in DHF Vietnamese children that a prolonged aPTT >30 s and platelet count <50 x 10^9^/L had an increased risk of bleeding [9]. They also suggested that thrombocytopenia is a mortality predictor in this population of patients [9]. The discrepancy between our findings and those reported by others could be explained, at least in part, by the differences in severity among DVI studied patients [2, 4].

Bleeding complications in DVI patients have been associated with a combination of thrombocytopenia, reduced thrombin formation and increased fibrinolysis [19, 20]. According to Nimmannitya, even extremely low platelet count, such as <20 x 10^9^/L, does not increase bleeding risk except in prolonged shock states [21]. This is a very interesting observation since DVI patients frequently exhibited low platelets in association with impaired platelets function [4, 7, 19]. Platelets are crucial for primary hemostasis as they contribute to thrombus formation [10, 22]. Fibrinogen, the final substrate of coagulation and the ligand of platelet glycoprotein IIb-IIIa complex receptors, enhance platelets function [10, 16]. Therefore, we can assume that the low frequency of bleeding manifestations in thrombocytopenic DVI patients in our study can be explained, at least in part, by maintained plasma fibrinogen levels, as shown in FIBTEM analysis [13, 15].

It is likely that fibrinogen plays a key role in keeping clot strength in DVI patients during the first days of disease [10, 20, 22]. Fibrinogen, the factor I of coagulation system, is the most important numerically and functionally coagulation factor [12, 22]. Fibrinogen represents approximately ninety percent of the total amount of plasmatic coagulation factors and it is the first coagulation factor to fall below a critical value during bleeding and hemodilution [10, 20, 22, 23]. Nevertheless, the critical plasma fibrinogen level associated to increased severity of DVI patients due to major bleeding events, hospitalization and death, needs to be determined [3, 4, 9, 20].

Disseminated intravascular coagulation (DIC) is defined by the presence of four criteria: thrombocytopenia (platelets below 100 x10^9^/L), high level of products of fibrin degradation (PDF), such as d-dimer, PT prolongation and low plasma fibrinogen level [24]. Our study demonstrated that patients in the first days of DVI already met at least two out of four criteria for DIC (low platelets and high PDF). Studies addressing hemostasis in DHF patients showed that all DHF patients manifested acute type of DIC [19, 20, 25]. Prolongation of aPTT and PT, decreased platelets count, plasma fibrinogen level, prothrombin, factor VIII, plasminogen and antithrombin III activities were observed transiently during acute phase of DHF [9, 20, 25] and they characterize hemorrhagic diathesis of DVI severe patients [3, 4, 20, 21, 25].

Viscoelastic tests allow early and individualized coagulation management in different scenarios, such as in trauma [26, 27], liver transplantation [13, 28], cardiac [15, 28] and neurologic surgeries [16, 29], post-partum hemorrhage [12, 27] and in critically ill patients [22, 26, 28]. Nevertheless, dengue treatment is based mainly on supportive care with fluids and electrolytes [2, 4, 30, 31]. Transfusion triggers and therapeutic goals are not consensus in DVI patients [2, 5, 30, 31]. A report of four DVI bleeding patients with severe DVI and thrombocytopenia in which desmopressin were administrated, showed clinical improvement and hemorrhage control [32]. Desmopressin is a hormone that stimulates release of Von Willebrand factor (vWF) by endothelial cells [10, 22]. The complex factor VIII (FVIII) and vWF improve platelets aggregation and clot stability [33]. Increased levels of vWF were demonstrated in DVI patients during the first days of disease [19, 33]. FVIII/vWF complex is likely to play a key hemostatic role during the early course of DVI [10, 22]. Endothelial activation may be responsible for extravascular plasma leakage and shock in severe DVI patients [2–5]. However, in the majority of DVI patients, inflammation and endothelial cell activation may represent a compensatory mechanism for thrombocytopenia, clot impairment and hypocoagulability during the early course of disease due to fibrinogen activation and increased vWF levels, which improve platelet aggregation [33–35].

Another key point in the treatment of DVI patients is the need of high I.V. volume expansion due to intense plasma leakage [2, 30, 31]. Thus, dilution coagulopathy may be present in DVI patients and further aggravate coagulation [2, 22, 34]. A thromboelastometry study showed clot impairment after fluid challenge infusions [36]. A fibrin polymerization deficit is assumed to be one of the major side effects of colloids and crystalloids on coagulation. However, impaired coagulation due to dilution coagulopathy is usually transient and can be partially reversed by fibrinogen concentrate transfusion [10, 36]. Moreover, colloids can also impair thrombus generation [22], decrease factor XIII-fibrin polymer interaction [36] and decrease platelet aggregability and adhesion [10, 11]. This could perpetuate a vicious circle of clot impairment in DVI patients with hypocoagulability secondary to a viral infection [37]. Therefore, we need to keep in mind the importance of individualizing volume expansion in DVI patients presenting with coagulopathy [31–34]. Therefore, a thromboelastometry-driven approach could represent an alternative strategy to manage complex cases of DVI associated with coagulopathy [37–39].

Our study has limitations. First, a small sample of DVI patients was included in this analysis. Nevertheless, to our knowledge, this was the first time DVI patients were analyzed with viscoelastic test such as thromboelastometry. Furthermore, most patients included in this report had primary DVI and were not severely ill, which might have affected and/or ameliorated their conventional coagulation tests results. Second, conventional coagulation tests abnormalities and bleeding disorders could be more pronounced in later stages during the course of disease and therefore not detected by our study. Third, dengue virus infection was confirmed by using immunochromatographic assays, which may lack sensitivity and specificity compared to real time polymerase chain reaction (PCR) or enzyme-linked immunosorbent assays (ELISA) for detection of IgM/IgG antibodies and NS1 antigen [40]. Finally, although Zika and Chikungunya viruses co-infections are well known today, they were unknown when this study was conducted [41]. Since diagnostic tools for Zika virus infection were not available in Brazil when the study was carried out, we cannot rule out Zika virus co-infection in our studied patients.

## Conclusion

Outpatients with dengue virus infection and thrombocytopenia commonly exhibited impaired thromboelastometry despite normal conventional coagulation tests. That suggests viscoelastic tests might have a higher sensitivity to detect early coagulation abnormalities in this population of patients. Long-term outcomes and the question of whether or not transfusion resuscitation algorithms based on viscoelastic tests will improve outcomes in DVI patients with coagulopathy and bleeding needs to be further evaluated.

## Abbreviations

A 10: Amplitude 10
aPTT: Activated partial thromboplastin time
CFT: Clot formation time
CT: Clotting time
DHF: Dengue hemorrhagic fever
DIC: Disseminated intravascular coagulation
DV: Dengue virus
DVI: Dengue virus infection
EXTEM: Extrinsically activated thromboelastometry test
FIBTEM: Fibrin-based thromboelastometry test
HR: Heart rate
INR: International normalized ratio
INTEM: Intrinsically activated thromboelastometry test
IQR: Interquartile range
IV: Intravenous
MCF: Maximum clot firmness
NS1: Non-structural protein-1
PDFs: Products of fibrin degradation
PT: Prothrombin time
ROTEM**®**: Rotational thromboelastometry test
SBP: Systolic blood pressure
SD: Standard deviation
TT: Thrombin time
vWF: Von Willebrand factor
